# The Benefits of Integrating Electronic Medical Record Systems Between Primary and Specialist Care Institutions: Mixed Methods Cohort Study

**DOI:** 10.2196/49363

**Published:** 2025-04-22

**Authors:** Kim Huat Goh, Adrian Yong Kwang Yeow, Le Wang, Hermione Poh, Hannah Jia Hui Ng, Gamaliel Tan, Soon Khai Wee, Er Luen Lim, Jared Louis Andre D’Souza

**Affiliations:** 1 Nanyang Technological University Singapore Singapore; 2 Singapore University of Social Sciences Singapore Singapore; 3 City University of Hong Kong Hong Kong China (Hong Kong); 4 National University Health System Singapore Singapore; 5 Ortho Spineworks Singapore Singapore; 6 National University Polyclinics Singapore Singapore; 7 National University Hospital Singapore Singapore

**Keywords:** EMR integration, primary care, specialist care, medical neighborhood, efficiency

## Abstract

**Background:**

The benefits of a fully integrated electronic medical record (EMR) system across primary and specialist care institutions have yet to be formally established. Integrating the EMR systems between primary and specialist care is the first step in building a medical neighborhood. A medical neighborhood is a set of policies and procedures implemented through integrated systems and processes that support the joint management of patient care across primary care physicians, specialist physicians, and other health care providers.

**Objective:**

This study aims to quantify the impacts of integrating the EMR systems of primary and specialist care institutions in the process of developing a medical neighborhood. The impacts are operationalized in both quantitative and qualitative measures, measuring the benefits of such an integration in 3 specific areas, namely, patient diagnosis tracking, patient care management, and patient coordination.

**Methods:**

A comprehensive, mixed methods examination was conducted using 3 different data sources (EMR consultation data, clinician survey data, and in-depth interviews). The EMR data consist of patient encounters referred to a specialist clinic from 6 primary care providers before and after integrating the EMR system into the primary and specialist care institutions. We analyzed 25,404 specialist consultation referrals to the specialist clinics by the primary care partners for a 12-month period, during which the integration of the EMR system was conducted. A cohort empirical investigation was used to identify the quantitative impacts of the EMR integration, and a follow-up survey was conducted with the clinicians 18 months post integration. The clinicians’ perceptions of the integration were measured to triangulate the empirical observation from the patient encounters, and the postimplementation perception survey was analyzed to triangulate the empirical investigation of consultation instances of the earlier cohort. Concurrently, a total of 30 interviews were conducted between March 16, 2021, and July 28, 2021, with clinicians and operations staff to gather on-the-ground sentiments engendered by this integration, which further informed our quantitative findings.

**Results:**

The integration of EMR systems between primary and specialist care institutions was associated with benefits in patient diagnosis tracking, patient care management, and patient coordination. Specifically, it was found that the integration resulted in a decrease in wait time for specialist appointments of an average of 16.5 days (*P*<.001). Patients were also subjected to fewer repeated procedures and tests; the number of procedures (*P*=.006), radiographies (*P*=.02), and overall bill sizes (*P*=.004) all decreased by between 4.08% and 39.7%, resulting in reduced health care resource wastage while maintaining similar medical outcomes (*P*=.37).

**Conclusions:**

Our study’s results are among the first instances of empirical evidence to show that the integration and sharing of data between primary and specialist care institutions promote continuity in health care delivery and joint patient management in a medical neighborhood. The findings go beyond the traditional benefits of improved referral communication, as shown in prior literature.

## Introduction

The problem of patient referral and coordination between primary and specialist care continues to be an ongoing challenge. In 2005, the average primary care physician (PCP) in the United States needed to coordinate patient care with 229 other physicians in as many as 117 practices [1], and such figures are representative of health systems where the aging population and number of health care providers are growing in tandem. However, such an interinstitutional fragmentation of care, as shown in a literature review published in 2020 [2], is associated with poorer health outcomes. The US Department of Health and Human Services defines care coordination as “the deliberate organizing [of] patient care activities and sharing [of] information among all of the participants concerned with a patient’s care to achieve safer and more effective care” and identified health information technology as a critical resource in this endeavor. Despite the need for close coordination among different health care providers, IT-enabled communication between primary and specialist care institutions is limited, which has negatively impacted patient care continuity [3].

Prior studies and the American College of Physicians have proposed the patient-centered medical neighborhood (PCMH-N) framework to promote coordinated care between PCPs and other providers for a given group of patients [4-8]. A medical neighborhood essentially involves a set of policies and procedures implemented through integrated systems and processes that support the joint management of patient care across PCPs, specialist physicians, and other health care providers [9,10]. If implemented correctly, PCMH-N will lead to faster and more appropriate referrals for specialist care with less wastage and repeated medical diagnostics, and as a result, patients can expect better care outcomes [3,9,11,12].

In Fisher’s [4] seminal work, 5 components were identified and weighted in terms of their importance in the implementation of PCMH-N; among them, the use of integrated health IT systems for *patient diagnosis tracking* (ie, to have synchronous electronic medical record [EMR] systems track patients’ diagnoses, test results, prescriptions, etc, across different institutions) is ranked top and accounts for 50% of the total resources required. Systems and guidelines to ensure *patient* *care management* and *patient coordination* (referrals across institutions) are ranked second, which accounts for 15% of the resources required. Developing communications standards, developing patient care plans, and performance reporting are the other 3 components that jointly account for the remaining 35% of the resources required. In this study, the focus was placed on measuring the aforementioned components that ranked most important in building a PCMH-N (ie, patient diagnosis tracking, patient care management, and patient coordination), and these measures were used to guide the study’s research design and data collection. The overall objective of this mixed methods study is to conduct an implementation assessment of the benefits of a fully integrated EMR system across different PCPs and specialist care health care institutions. We examined the benefits using 3 dimensions. First, in the area of *patient diagnosis tracking*, we measured whether the synchronous flow of complete patient clinical data among PCPs and specialists, as well as the communication among PCPs, specialists, and their administrative teams, would result in a reduction of repeated laboratory tests, diagnostic radiology tests, and overall patient bill sizes. Second, in the area of *patient care management*, we examined whether there were differences in patient outcomes and reductions in medical procedures due to the EMR integration. Third, in the area of *patient coordination*, we measured whether the EMR integration and standardized referral process improved tracking and follow-up among primary care clinics and specialist clinics thereby leading to shorter wait times for specialist appointments.

The purpose of this research is to measure the effects of integrating EMR systems between primary and specialist care institutions. To achieve this objective, three types of data were used: (1) patient encounter data from the EMR system (data source 1, DS 1), (2) clinician survey data (data source 2, DS 2), and (3) interviews (data source 3, DS 3). Our study aims to provide a comprehensive understanding of the benefits of a fully integrated EMR system across different health care institutions to develop a PCMH-N. The findings of this research can serve as a guide for other health care organizations on how to successfully implement an integrated system.

## Methods

### Data Collection and Data Sources

#### DS 1: EMR Data Collection

We collected data from a cohort of 25,404 patient encounters referred from 6 primary care clinics to various specialist clinics from April 1, 2020, to March 31, 2021. The inclusion criteria are all specialist care referrals from the 6 primary care clinics referred to the specialist clinic for the period. No sampling was performed to reduce sampling or selection biases. Each of these 6 primary care clinics typically handles about 800 consultations per clinic per day, of which there are instances where patients have to be referred to a specialist clinic. The data were extracted from the EMR system of the health institutions. Of these referrals, 22,309 have complete data; the remaining 3095 encounters have some missing data due to the patient not turning up for the appointment. The observations with some missing variables were listwise omitted from the analysis if the missing variables were part of the model specification, there was no imputation of missing data, and the missing rate was equivalent to institutions’ historical averages. Additional details of the collection methodology are reported using the STROBE (Strengthening the Reporting of Observational Studies in Epidemiology) guidelines and can be found in Multimedia Appendix 1.

Between September 26, 2020, and November 16, 2020, the primary care clinics changed to a new EMR system that was integrated with those in the specialist clinics. The integration involves synchronous, read-and-write access to the medical notes, patient scheduling, and viewing of all patient medical records, including laboratory tests, radiographies, and so forth, by both the PCPs and specialist care clinicians. As a result, the referral process for the primary care clinics was standardized in the EMR system. The EMR integration was performed on a weekend when the consultations for the weekend were suspended. A total of 14,124 encounters in the specialist clinics occurred before the integration, and 11,280 encounters occurred after the integration.

#### DS 2: Clinician Survey

As suggested in the literature [13], we surveyed clinicians in both primary and specialist care institutions to gain insights into the factors potentially driving any difference in the management of referrals. The survey was emailed to all clinicians working in primary and specialist care clinics in June 2022 who are impacted by the integration of the EMR system. A total of approximately 200 clinicians were emailed. Due to the confidentiality agreement with the health care institutions, these emails were sent to the clinicians via the hospital network and not by the researchers. A total of 104 clinicians replied to the survey (81 from the primary care clinics and 23 from the specialist clinics) at a 95% CI; the margin of error for the survey was 6.7%. The survey responses were anonymized, and all responses were retained for analysis and reporting.

#### DS 3: In-Depth Interviews

In addition to the survey, the researchers also conducted in-depth qualitative interviews with different stakeholders. The data collection period was from March 16, 2021, to July 28, 2021. Interviewees were selected based on purposive sampling to have a diverse set of interviewees involved in the referral process. As such, we selected interviewees from (1) those who worked in the specific specialist clinics (chosen for the high volume of referrals received from the primary care clinics), (2) the call center, and (3) those in specific primary care clinics (chosen for sending high number of referrals to the specialist clinics). Within those clinics and call centers, we selected those who held clinical and nonclinical roles and were involved in the referral process (from writing the referral to processing, scheduling, and receiving the referrals). As our goal was to understand the perceptions of the clinicians and admin staff concerning the EMR integration and its impact on the referral process, invitations for interviews were sent via email more than 3 months after the integration. This would provide a more balanced perception of the integration after a significant period of use. A total of 30 interviews were conducted—15 at the specialist clinic and call center (clinicians, clinic managers, and call center staff), and 15 at the primary care clinics (PCPs, managers, and referral admin staff). Refer to Table 1 for the breakdown of the interviews by the interviewees’ respective roles and organizations.

Interviews were conducted either at the interviewees’ office or via web-based mode (Zoom; Zoom Video Communications, Inc). The duration of each interview was between 30 minutes and an hour and they were conducted by 2 interviewers (AYKY and CLGB). A set of standardized interview questions was used, but the interviewers allowed the interviewee to guide them based on what they felt was more salient based on their experience. Most of the interviews had audio recordings and were transcribed after each interview. For those that were not recorded, copious notes were written. Additional details of the interview methodology are given in Multimedia Appendix 2 using the COREQ (Consolidated Criteria for Reporting Qualitative Research) checklist.

**Table 1:** Number of interviews by roles and organization^a^.

Roles and organization			Interviews post-EMR^b^ integration, n
**Specialist clinics**			
	Specialists (and specialist nurses)	9	
	Clinic manager	2	
	Call center admin staff	4	
	Subtotal	15	
**Primary care clinics**			
	PCPs^c^	6	
	Manager	3	
	Referral admin staff	6	
	Subtotal	15	
Total			30

^a^Each count represents an interview instance with a unique staff member lasting between 30 and 60 minutes. Refer to Multimedia Appendix 2 for more details about the selection methodology.

^b^EMR: electronic medical record.

^c^PCP: primary care physician.

### Analysis of Data Collected

#### DS 1: Analyzing EMR Data

We collected details relating to all the referral patient encounters between the 6 primary care clinics and the specialist care clinic. The unit of analysis for the estimation is per patient referral encounter, *j*, where

y_ij_ = *f*(*X_ij_,Z_ij_,U_i_*)

where *y* represents 6 continuous dependent variables and 1 binary dependent variable. *X* represents the vector of control variables, *Z* is a binary variable representing pre- and post-EMR integration, *U_i_* represents the specific error term for each primary care clinic, and *i* and *ij* represent each patient encounter.

The outcome variables are represented as *y*. The 6 continuous dependent variables were measured to operationalize the benefits of patient diagnosis tracking, care management, and coordination. These dependent variables were (1) the patient’s wait time for a specialist appointment (patient coordination), (2) the number of procedures conducted at a specialist clinic (patient care management), (3) the number of panel tests conducted at a specialist clinic (patient diagnosis tracking), (4) the number of laboratory tests conducted at a specialist clinic (patient diagnosis tracking), (5) the number of radiographies conducted at a specialist clinic (patient diagnosis tracking), and (6) the bill size of the specialist consultation (patient diagnosis tracking). Due to confidentiality reasons, bill sizes were standardized by dividing individual bill encounter sizes over the overall mean bill size of the dataset. If improved patient diagnosis tracking occurred, all else held constant, a reduction in the number of radiographies, laboratory tests, and overall bill sizes in the specialist clinics was expected, given that these reports from the primary care clinics had been made available to the specialists via the EMR integration. To measure whether health care outcomes (patient care management) had changed post-EMR integration, the dependent variable of the resolution (or lack thereof) of the clinical encounter was recorded as a binary outcome.

The control variables, *X*, for this estimation included medical specialty, the *International Classification of Diseases, Tenth Revision* (*ICD-10*) problem code, the procedure performed, the case’s urgency as assessed by the PCP, and the patient’s gender, race, marital status, and visit date. These variables partialed out the majority of the confounding factors that would impact the referral process.

We used both fixed-effects feasible generalized least squares and random-effects feasible generalized least squares estimators to analyze the relationship between the 6 outcome variables represented by *Y*, the independent variables represented by *X* and *Z* for all the referral encounters from April 1, 2020, to March 31, 2021. This methodology accounts for unobserved, time-invariant heterogeneity by including unit-specific intercepts and addresses potential heteroscedasticity or serial correlation in the error terms. Specifically, the estimation procedure involves two steps: (1) fixed effects (and random effects) are initially estimated using ordinary least squares, and (2) residuals from the first step are used to construct a feasible generalized least squares weighting matrix for a second-step estimation. Robust SEs are reported to account for possible heteroskedasticity of the data.

For fixed-effects and random-effects estimations, we used the grouping variable of each primary care clinic to account for time-invariant primary care clinic characteristics and the source of the primary care clinic referral. The estimation was conducted in Stata 18 (StataCorp LLC) using the XTREG function for all 6 fixed-effects and 6 random-effects feasible generalized least squares [14]. These would help us estimate the posteffects of the EMR integration for the medical neighborhood.

Finally, we also performed 1 fixed-effects and 1 random-effects logistic regression [15] to estimate the inline graphic 1 specification with the binary dependent variable of whether the clinical encounters were resolved. The estimation was conducted in Stata 18 using the XTLOGIT function. Likewise, the results were reported according to the STROBE guidelines, as documented in Multimedia Appendix 1.

#### DS 2: Analyzing Survey Data

To triangulate our empirical results from the consultation records [16], we conducted a secondary analysis to measure the perceptions of both primary and specialist care clinicians in five areas, specifically (1) their access to clinical notes, (2) their access to test results, (3) referral appointment scheduling, (4) referral management, and (5) quality of care. Items 1 and 2 measured patient diagnosis tracking, items 3 and 4 measured patient coordination, and item 5 measured patient care management.

For each category, we surveyed clinicians 18 months postimplementation to gauge their sentiments about the possible changes brought about by the EMR integration. PCPs and specialists were asked to rate (on a 5-point scale) [17] whether the EMR integration for the PCMH-N presented changes to their daily work in each area. The questions were edited to fit their primary care or specialist care contexts.

#### DS 3: Analyzing Interview Data

We used qualitative content analysis to analyze the interview data collected after the implementation of the EMR system [18]. To have a better comparison between the 2 key stakeholders, the analysis was divided into primary care clinic and specialist clinic data. We compared the themes emerging from the primary care clinics’ data with those from the specialist clinics’ data to observe their similarities and differences. The coding of interview data was conducted by 1 researcher (AYKY) and validated by a research associate (CLGB). All of the interview data were coded using MaxQDA (version 2020; VERBI GmbH). Overall, our coding analysis generated themes that we mapped to our survey, that is, access to referral notes and tests, referral scheduling and management, and overall quality of care. Furthermore, we surfaced important differences between the PCPs’ and specialists’ perceptions within some of the themes. Interview texts were included in the “Results” section to illustrate these themes.

### Ethical Considerations

The ethics approval for this study was obtained on September 8, 2020, from the National Healthcare Group, Domain Specific Review Board, Singapore (National Healthcare Group, Domain Specific Review Board reference no. 2020/00156). This approval covered all 3 data sources. Informed consent for data sources DS 2 and DS 3 were explicitly obtained. For DS 1, under the Human Biomedical Research Act 2015, informed consent is deemed to be obtained for deidentified, archival medical records. All data collected were deidentified, and human subjects were not compensated for participating in this research.

## Results

### Overview

Both sets of random- and fixed-effects estimation yielded similar results. For brevity, unless specified otherwise, we described the findings using the fixed-effects estimation (Tables 2 and 3).

**Table 2:** Results from fixed-effects estimation for specialist clinic referrals^a^.

Dependent Variable	Construct	Fixed effects, FGLS^b^ estimation				
		Coefficient	SE	*P* value	95% CI	*R* ^2^
Wait time (days) to referral appointment date	Patient coordination	–16.4933	0.7257	<.001	–18.3587 to –14.6280	0.335
Number of procedures at specialist clinic	Patient care management	–0.0398	0.0086	.006	–0.0618 to –0.0178	0.292
Number of panel tests at specialist clinic	Patient diagnosis tracking	–0.0159	0.0092	.14	–0.0395 to 0.0076	0.403
Number of laboratory tests at specialist clinic	Patient diagnosis tracking	–0.0064	0.0130	.64	–0.0398 to 0.0270	0.445
Number of radiographies at specialist clinic	Patient diagnosis tracking	–0.0408	0.0125	.02	–0.0730 to –0.0085	0.274
Bill size (index dollars)	Patient diagnosis tracking	–0.0780	0.0156	.004	–0.1181 to –0.0379	0.532
Condition resolved	Patient care management	–0.0973	0.1092	.37	–0.3113 to 0.1168	N/A^c^

^a^We anonymized the actual bill size for confidentiality reasons; the consultation bill sizes were divided by an index value—coefficients represent the drop in percentage of the bill size. Fixed- and random-effects ordinary least squares estimations were also performed with similar statistical results but not reported due to data heteroskedasticity.

^b^FGLS: feasible generalized least squares.

^c^N/A: not applicable.

**Table 3:** Results from random-effects estimation for specialist clinic referrals^a^.

Dependent variable	Construct	Random effects, FGLS^b^ estimation					
		Coefficient	SE	*P* value	95% CI	*R* ^2^	
Wait time (days) to referral appointment date	Patient coordination	–16.2349	0.6235	<.001	–17.4568 to –15.0129	0.335	
Number of procedures at specialist clinic	Patient care management	–0.0392	0.0083	<.001	–0.0555 to –0.0229	0.292	
Number of panel tests at specialist clinic	Patient diagnosis tracking	–0.0164	0.0086	.06	–0.0333 to 0.0005	0.403	
Number of laboratory tests at specialist clinic	Patient diagnosis tracking	–0.0065	0.0130	.62	–0.0319 to 0.0189	0.445	
Number of radiographies at specialist clinic	Patient diagnosis tracking	–0.0409	0.0126	<.001	–0.0656 to –0.0162	0.275	
Bill size (index dollars)	Patient diagnosis tracking	–0.0787	0.0148	<.001	–0.1078 to –0.0496	0.532	
Condition resolved	Patient care management	–0.0507	0.1173	.67	–0.2806 to 0.1792	N/A^c^	

^a^We anonymized the actual bill size for confidentiality reasons; the consultation bill sizes were divided by an index value—coefficients represent the drop in percentage of the bill size. Fixed-effects and random-effects ordinary least squares estimations were also performed with similar statistical results but not reported due to data heteroskedasticity.

^b^FGLS: feasible generalized least squares.

^c^N/A: not applicable.

In terms of *patient diagnosis tracking*, we observed some improvements in patient tracking through the EMR integration. First, we observed a significant reduction in the number of radiographies conducted after the integration (coefficient –0.0408; *P*=.02). This translates to a 39.6% decrease in the number of radiographies post integration when the coefficient is divided by the mean number of radiographies conducted for all procedures. The synchronous availability of radiographic images and reports between primary and specialist care institutions perhaps reduced the need for specialist clinics to perform repeated radiographies.

Intuitively, with the reduction in the number of procedures and radiographies, the regressions showed an overall decrease of 7.8% in bill size after the EMR integration (*P*=.004). The integration, however, did not bring about a reduction in resource use in other areas. Specifically, there were no changes observed in the number of laboratory tests conducted at the specialist clinics after the integration (*P*=.64). Similarly, the estimation results provided minimal statistical support for a reduction in the number of panel tests conducted after the EMR integration (fixed-effects coefficient –0.0159, *P*=.14; random-effects coefficient –0.0164, *P*=.06). We discuss some of the possible reasons for these results in the Discussion section.

In terms of *patient care management*, a significant drop was observed in the number of procedures conducted at the specialist clinic (coefficient –0.0398, *P*=.006, which translates to a 30.4% drop in procedures). This is calculated by dividing the drop by the mean number of procedures for all consultations by 0.131. Similarly, no changes were observed in the number of encounters marked as resolved after the consultation (*P*=.37), suggesting no significant aggregated change in medical outcomes after integrating the primary and specialist care EMR systems.

Finally, our results showed improvements in *patient coordination*. Based on the descriptive statistics (Table 4), we observed that the mean wait time was shortened from 34.65 days to 21.03 days over 12 months, during which the EMR was integrated between the primary and specialist care clinics (Table 5). The FLGS estimation (Table 4) also showed that the average wait time for a referral from primary to specialist care decreased by 16.49 days (*P*<.001) after the EMR integration.

**Table 4:** Table4. Descriptive statistics of dependent variables from electronic medical record system (before and after integration).

Variable	Construct	Before integration			After integration	
		Mean (SD)	Median (IQR)	Mean (SD)		Median (IQR)
Wait time (days) to referral appointment date	Patient coordination	34.651 (32.352)	26 (8-52)	21.040 (19.970)		13 (5-35)
Number of procedures at specialist clinic	Patient care management	0.122 (0.384)	0 (0-0)	0.146 (0.411)		0 (0-0)
Number of panel tests at specialist clinic	Patient diagnosis tracking	0.101 (0.520)	0 (0-0)	0.100 (0.540)		0 (0-0)
Number of laboratory tests at specialist clinic	Patient diagnosis tracking	0.068 (0.411)	0 (0-0)	0.068 (0.417)		0 (0-0)
Number of radiographies at specialist clinic	Patient diagnosis tracking	0.100 (0.416)	0 (0-0)	0.108 (0.423)		0 (0-0)
Bill size (index dollars)	Diagnosis tracking	0.998 (0.750)	0.717 (0.611-1.130)	1.011 (0.710)		0.717 (0.611-1.164)
Condition resolved	Patient care management	0.044 (0.205)	0 (0-0)	0.045 (0.207)		0 (0-0)

**Table 5:** Descriptive statistics of dependent variables from the electronic medical record system.

Variable	Construct	Overall	
		Mean (SD)	Median (IQR)
Wait time (days) to referral appointment date	Patient coordination	29.775 (29.277)	20 (7-46)
Number of procedures at specialist clinic	Patient care management	0.131 (0.394)	0 (0-0)
Number of panel tests at specialist clinic	Patient diagnosis tracking	0.101 (0.527)	0 (0-0)
Number of laboratory tests at specialist clinic	Patient diagnosis tracking	0.068 (0.413)	0 (0-0)
Number of radiographies at specialist clinic	Patient diagnosis tracking	0.103 (0.419)	0 (0-0)
Bill size (index dollars)	Diagnosis tracking	1.000 (0.743)	0.717 (0.611-1.137)
Condition resolved	Patient care management	0.045 (0.206)	0 (0-0)

### Survey and Interview Results

We analyzed the survey responses and interview data to triangulate the findings from the consultation records with the clinicians’ sentiments on the ground. Table 6 provides the survey results, where sentiment scores larger than 3 represent the positive change resulting from the integration, and values less than 3 present negative changes from the integration.

**Table 6:** Results from survey post–electronic medical record integration for medical neighborhood^a^.

Category			Construct		Questions		Mean (SD)		*P* value		95% CI
**Primary care clinicians**											
	Access to clinical notes	Patient diagnosis tracking		I find that it is easier to access patient notes that (the specialist) clinicians have written (after the integration).		3.90 (0.12)		<.001		3.65-4.15	
	Access to test results	Patient diagnosis tracking		I find that it is easier to access laboratory tests and medication (after the integration).		3.33 (0.20)		.10		2.94-3.73	
	Referral scheduling	Patient coordination		I observed that my clinic’s patient could get his or her scheduled appointment at (the specialist clinic) at an earlier date.		2.94 (0.14)		.69		2.66-3.22	
	Referral management	Patient coordination		My work of managing patient referrals from specialists benefits from my access to (specialist) clinicians’ patient notes and laboratory tests.		3.68 (0.18)		<.001		3.31-4.04	
**Specialist care clinicians**											
	Quality of care	Patient care management		I believe that (the integration) has enabled my clinic to provide better clinical care for my patients referred to (specialist care).		3.37 (0.15)		.02		3.06-3.68	
	Access to clinical notes	Patient diagnosis tracking		Since (the integration), I feel that it is easier to access referral notes and patient notes written by the (primary care) clinicians and referral staff.		3.79 (0.26)		.009		3.23-4.35	
	Access to test results	Patient diagnosis tracking		Since (the integration), I feel that it is easier to access a (primary care) referral patient’s laboratory tests and medication lists.		4.23 (0.17)		<.001		3.89-4.59	
	Referral scheduling	Patient coordination		Since (the integration), I observed that (primary care) referral patients had their appointment booked at an earlier date.		3.92 (0.23)		.002		3.41-4.42	
	Referral management	Patient coordination		Since (the integration), I feel that accessing (primary care) referrals has become less time-consuming.		3.50 (0.23)		.048		3.01-3.99	
	Quality of care	Patient care management		(The integration) has helped me to improve overall clinical care and service to (primary care) referral patient.		4.11 (0.20)		<.001		3.65-4.57	

^a^Measures from a scale of 1-5, where 3 represents the midpoint where no change was perceived after the integration. Values larger than 3 represent a positive perception toward the integration, and values less than 3 represent a negative perception toward the integration. Two-sided *P* values and 95% CIs are reported.

#### Patient Diagnosis Tracking

PCPs and specialists agreed that the EMR integration brought about greater accessibility to clinical notes, where they could read the details of the notes written by clinicians across primary and specialist care institutions (primary care 3.90, SD 0.12, *P*<.001; specialist care 3.79, SD 0.26, *P*=.009). This sentiment was reflected in a quote from a PCP who said:

I think it’s easier to give a referral. They can look at our notes, and we can look at their notes—regarding what happened, what their discussion was. So, I think that’s actually a convenience.

A specialist concurred, saying:

Since we can access the notes, it is better than the previous time, as we can see what they have assessed. They would have enquired about their past medical history and drug allergies, and the reason for referral is also much clearer than before.

Likewise, PCPs found marginal improvement in the accessibility of standardized records such as laboratory tests (mean 3.33, SD 0.2; *P*=.10), while the specialists perceived significant improvements in the accessibility of these standardized records (mean 4.23, SD 0.17; *P*<.001). This improvement in notes also aided the specialist during patient examination:

Yes, it makes it more convenient (and) uniform, and I know that patient has a heart problem, a kidney problem, I can just further enquire, “what medications are you taking?” rather than going all over again “do you have any heart problem?”

Orthopedic Specialist

#### Patient Coordination

In terms of referral scheduling and referral management, we observed that PCPs did not perceive any positive changes in referral scheduling (mean 2.94, SD 0.14; *P*=.69) but observed improved ease in referral management (mean 3.68, SD 0.18; *P*<.001). The admin staff explained how the referral management improved for patients:

So now we are on the same platform—I see all of the patient’s appointments, whether it’s in the [primary care clinic] or the [specialist clinic]. It is good because when we schedule an appointment, we try to avoid the date the patient has to go to the [primary care clinic], or it helps us understand where the patients are going on different days.

The specialists also observed that referral scheduling became faster (mean 3.92, SD 0.23; *P*=.002) and better managed (mean 3.5, SD 0.23; *P*=.048). This is reflected by a specialist clinic staff who said, “Efficiency increased and productivity (of referrals) increased, because (we) don’t need to toggle between multiple applications to handle one referral.”

#### Patient Care Management

Finally, both PCPs and specialists felt that the quality of care for the patients after the integration had improved (primary care 3.37, SD 0.15, *P*=.02; specialist care 4.11, SD 0.2, *P*<.001). A PCP shared how the EMR integration helped with improving the quality of care:

I think the most relevant is the Cardio side, because they are running a clinic called the Rapid Access Chest Pain Clinic. So, usually, what we do is refer the patient to [the specialist clinic] for a very quick cardiac assessment. So, if they did the treadmill, the blood test comes back all normal, they will send [them] back to us to see. In this scenario, because we can see the Cardiology notes and their screening,..., we can more easily follow up for these patients.

## Discussion

### Principal Results

In this study, we triangulated the effects associated with EMR system integration using patient encounter records, survey, and interview data. The survey and interview results provided some explanations for our observations based on the quantitative outcomes associated with integrating primary and specialist care institutions. Table 7 presents an integrated summary of this study’s findings by juxtaposing the 3 dimensions of impact (ie, patient diagnosis tracking, patient care management, and patient coordination) resulting from the EMR integration, with the data sources and analysis from this study.

**Table 7:** Summary of findings^a^.

Data source	Methodology	Impact measured		
		Patient diagnosis tracking	Patient care management	Patient coordination
DS^b^ 1: Objective, patient encounter records	Cohort empirical investigation	Improvements in 2 of 4 measures	Improvements in 1 of 2 measures	Improvement in 1 of 1 measure
DS 2: Perception, survey	Survey of clinicians	Improvement observed in 3 of 4 measures	Improvement perceived in 2 of 2 measures	Improved perceived in 3 of 4 measures
DS 3: Perception, clinicians	In-depth interviews with clinicians	Improvement perceived by PCP^c^ and specialists	Improvement perceived by PCP and specialists	Improvement perceived by staff doing patient scheduling

^a^Quantitative findings are based on the different measures used to operationalize the 3 constructs of patient diagnosis tracking, patient care management, and patient coordination. The qualitative findings from the in-depth interviews are juxtaposed with the quantitative findings.

^b^DS: data source.

^c^PCP: primary care physician.

Regarding patient diagnosis tracking, from the survey and interviews, both PCPs and specialists found that it became easier to access the clinical notes written by clinicians from other institutions after the EMR system integration. This sentiment was quantitatively supported by our cohort’s empirical investigation, as seen in the reduced number of examinations and treatment procedures conducted by the specialists during their consultations.

Interestingly, in terms of the clinicians’ perception regarding the improved accessibility of standardized test results (eg, radiographies and laboratory tests), it was strongly felt by the specialists but less so by the PCPs. One possible reason for this is specialists may tend to read the PCPs’ initial diagnosis during the consultation when patients are referred to them. In our empirical estimation, we observed that the number of radiographies decreased after the EMR integration, while laboratory panel tests saw a marginal decline, and stand-alone laboratory tests did not experience any decline after the EMR integration. Our interview data suggested that specialists required laboratory tests to get more current readings from their patients, hence increasing the likelihood of conducting a repeated test during the specialist consultation, while specialists typically did not require additional radiographies if there were no material changes in the patient’s condition.

The reduced number of radiographies and procedures conducted in specialist clinics without any additional increase in other forms of diagnosis was naturally associated with an overall drop in the patient consultation bill size. As clinicians do not perform the patient’s billing and it is an objective quantifiable measure, hence, we did not survey the clinicians concerning this aspect post-EMR implementation. An overall lower bill for the patient represents a more efficient use of medical resources in health care systems.

From the patient encounter records that measured patient care management quantitatively, we observed a drop in the number of procedures performed, with no associated drop in the resolution rate of diseases. Although we did not see an improvement in the resolution rate from the objective data, the clinician survey results suggested an improvement in the perceived quality of care provided. We believe that the mixed results are due to the many definitions and measurements of the quality of care. Hence, we argue that perhaps care improvements cannot be fully measured with just the resolution rate of clinical problems. Instead, by adopting the clinicians’ broader perspective, we could observe some improvements in patient care after EMR integration. Taking these results together, a conservative conclusion is that the patient quality of care did not decline after the EMR system integration between primary and specialist care institutions. This finding is important as EMR system implementations will impact clinical workflow processes, and it is essential to ensure that the integration does not unintentionally compromise the quality of care.

Finally, from the patient’s perspective regarding care coordination post-EMR integration, our results show that they did experience shorter waiting times, as measured by the time required to schedule a specialist appointment. This quantitative finding was supported by the specialist clinic’s perception of the improvements seen in managing referrals and appointment scheduling [19]. This perceived improvement, however, is less salient for the PCPs who did not perceive a significant improvement in appointment scheduling. From our interviews with the PCPs, we discovered that one of the reasons behind this finding was that their call center staff typically handles the appointment schedule for PCP referrals. As such, PCPs may not be aware of their patient’s wait time for the next specialist appointment, which might explain their lack of perceived improvements post-EMR integration.

### Comparison With Prior Work

This study is one of few studies that empirically quantifies the benefits of synchronizing the EMR systems between primary and specialist care to form a medical neighborhood. This contributes to the existing medical neighborhood literature, as most of those studies were mainly advocating for the medical neighborhood through observational and logical deduction [3,4,9,11], while our study provides empirical evidence to support the medical neighborhood framework.

Furthermore, our research extends existing research in 3 key areas. First, prior studies that examined the referral integration between primary and specialized care institutions tend to study those where there is an asynchronous sharing of data between the PCPs and specialists [20] (ie, the PCP provides patient medical data in their referral to the specialist, but the specialist does not share patient medical data back to the PCP, which prevents the comanagement of the patient [9]). This asynchronous sharing is mainly due to the fact that the referral systems used essentially functioned like messaging systems between primary and specialist care institutions and they did not involve a full EMR integration between primary and specialist care, which would have enabled the 2-way referral information sharing [21]. Our investigation involved synchronous sharing and integration of medical records as part of referral integration, thereby permitting a closed referral loop.

Second, the outcomes of the integration of such referral systems are often mixed [22]. Some studies showed that integration reduced medical costs based on claims and fewer specialist visits [20]. Others found an increase in specialist referrals due to potentially tighter integration between primary and specialist care [23]. Regarding care coordination, referral systems were associated with poor information integration across stakeholders [24] but helped to improve wait time, communication, and administrative efficiencies [19]. Other studies suggested that the integration of EMR systems reduced unnecessary laboratory testing and imaging when previous test results were made available to referral specialists [25]. Many of these studies examined referral integration using a single data source and methodology, which presented a significant albeit limited view of integration. In contrast, our study provided a comprehensive examination of integration, showing concrete benefits by using multiple data sources to triangulate our results.

Third, most existing research relied on quantitative or qualitative indicators to examine referral and integration impacts. However, to properly assess the outcomes of EMR integration in PCMH-N, scholars have proposed that we should leverage a mix of quantitative indicators (eg, electronic tracking of referrals) and qualitative measures from clinician surveys and in-depth interviews (cf Tables 2 and 3 [9]). Specifically, Greenberg et al [9] called for a more holistic approach to measuring the performance of medical neighborhoods using EMR data, along with surveying and interviewing clinicians. Likewise, Vimalananda et al [13] suggested surveying clinicians to better understand what the success of such an implementation entails since the success of the medical neighborhood relies on the complicated alignment and coordination between PCPs and specialists [3]. We heed these calls for a more holistic assessment by using a multimethod approach in our research inquiry.

In sum, this work provides an initial step toward the validation of PCMH-N. The integration of EMR systems between primary and specialist care institutions is a necessary first step toward building a PCHH-N. Our literature review reveals that a comprehensive, empirical validation of the PCMH-N’s benefits is relatively scarce, with those that are focused on parts of the PCMH-N having mixed results [20,22,23]. The seminal PCMH-N papers are commentaries or observational studies that logically argue for PCMH-N’s benefits to public health and the management of health care resources [3,4,9]. Among the handful of studies that validated the PCMH-N’s benefits is the study by Tuot et al [11] where they found that a feedback ratings system in an e-referral system created high-quality PCP specialist preconsultative engagement and virtual comanagement of patients. Another study found that the use of an e-referral system for PCPs improved the ability to track referrals, reduced wait time for scheduled visits, and thereby improved patient access [26]. While these findings establish some aspects of the PCMH-N, they are based on asynchronous information sharing using an electronic messaging system and not through a fully integrated EMR system [12]. As such, many of the key benefits, such as reduced repeat testing and imaging [25] and comanagement [27] that could be delivered by the PCMH-N, are still not extensively explored. Our study presents the findings of an integration that has fully synchronous medical data sharing across all institutions.

### Limitations

As in all surveys and interviews, participation is voluntary. Although we have conducted an analysis and confirmed that early and late respondents have statistically similar responses, the possibility of respondent self-selection biases for the survey data exists.

In addition, given the multimethod approach used in this study, the main purpose of the survey results is to triangulate the findings from the quantitative EMR data. The survey questions complemented the in-depth interviews. As a result, the questions used in the survey were not developed and validated in the typical comprehensive manner for survey scale development. The task of scale development can be a future study for researchers to embark on.

### Conclusions

The aim of integrating the EMR systems of PCPs and specialists is to improve care and coordination continuity for a specific patient community. Such EMR integration of primary and specialist care institutions could improve patient diagnosis tracking, patient care management, and patient coordination. These improvements are manifested as tangible benefits such as reduced diagnostic redundancy, shorter patient wait time for specialist appointments, and lower patient bill sizes without compromising health outcomes. In this study, we analyzed specialist referral patient encounters and surveyed clinicians from primary and specialist care clinics to provide a set of comprehensive empirical evidence of integration benefits. We further triangulated our findings with in-depth interviews with the clinicians to better uncover the nuances of these benefits. Our results support the move toward integrating primary and specialist care EMR systems for more efficient utilization of health care resources, which will particularly benefit health care systems that face significant resource constraints.

## Appendix

Multimedia Appendix 1 STROBE (Strengthening the Reporting of Observational Studies in Epidemiology) checklist.

Multimedia Appendix 2 COREQ (Consolidated Criteria for Reporting Qualitative Research) checklist.

## Abbreviations

COREQ: Consolidated Criteria for Reporting Qualitative Research
EMR: electronic medical record
PCMH-N: patient-centered medical neighborhood
PCP: primary care physician
STROBE: Strengthening the Reporting of Observational Studies in Epidemiology
