# Supplementary material for: The Benefits of Integrating Electronic Medical Record Systems Between Primary and Specialist Care Institutions: Mixed Methods Cohort Study
Source: J Med Internet Res. 2025 Apr 22;27:e49363. doi: 10.2196/49363 (PMC12056414; doi:10.2196/49363)
Supplement: Multimedia Appendix 1 [file jmir_v27i1e49363_app1.pdf]

## Appendix 1: STROBE – Methods & Results Reporting for Cohort Studies

| | Recommendation | Details |
|---|---|---|
| **Methods** | | |
| Study design | Present key elements of study design early in the paper | Study design and setting presented in the paper |
| Setting | Describe the setting, locations, and relevant dates, including periods of recruitment, exposure, follow-up, and data collection | From 1 April 2020 to 31 March 2021, all patient encounters in various specialist care clinics within Ng Teng Fong General Hospitals, Singapore were referred from six primary care clinics. |
| Participants | (*a*) Give the eligibility criteria, and the sources and methods of selection of participants. Describe methods of follow-up<br>(*b*) For matched studies, give matching criteria and number of exposed and unexposed | Inclusion criteria: Specialist care encounters from the six primary care clinics between 1 April 2020 and 31 March 2021.<br><br>Not applicable |
| Variables | Clearly define all outcomes, exposures, predictors, potential confounders, and effect modifiers. Give diagnostic criteria, if applicable | Outcome variables:<br>1. Patient wait time for specialist appointment<br>2. Number of procedure conducted at specialist clinic<br>3. Number of panel test conducted at specialist clinic<br>4. Number of lab tests conducted at specialist clinic<br>5. Number of X-rays conducted at specialist clinic<br>6. Bill size of the specialist encounter (normalized values for confidentiality reasons)<br>7. Binary flag to indicate if patient's ailment is resolved.<br><br>Covariates (Control Variables):<br>Medical specialty of the specialist clinic, the ICD-10 problem code, the procedure performed, the case's urgency as assessed by the primary care, the patient's gender, race, marital status, and visit date |
| Data sources/ measurement | For each variable of interest, give sources of data and details of methods of assessment (measurement). Describe comparability of assessment methods if there is more than one group | The unit of data extracted is each patient encounter identified by a patient ID and encounter ID extracted from the electronic medical records from the EPIC system used by the healthcare institutions.<br><br>All variables used in the analyses were obtained from these patient encounters. |
| Bias | Describe any efforts to address potential sources of bias | Given that the entire population of encounters was analysed, we do not expect any sampling bias for the patient encounter data. However, patient self-selection biases exist as we cannot analyze the difference between patients who select to go to these public healthcare institutions versus other patients who self-select into more costly private healthcare institutions. We recognize this as a limitation. |

| | | |
|---|---|---|
| Study size | Explain how the study size was arrived at | The study size was a function of the selection of the time frame for the patient cohort. As we hope to examine the effects of EMR integration, we included all specialist clinic encounters approximately six months before and six months after the EMR integration.<br><br>Given all encounters were analyzed, the sample size is deemed to be sufficient prior to the data collection as the clinics typically get about 25,000 encounters referred to them from the primary care clinics within our sample. |
| Quantitative variables | Explain how quantitative variables were handled in the analyses. If applicable, describe which groupings were chosen and why | We deliberately choose quantitative variables that were objective in this study for this cohort. All variables selected here captured the actual suite of activities that occurred during the patient encounter. We did not use any subjective measure for this particular dataset.<br><br>In the estimation, we group (and flag) the patient encounters that occurred prior to the EMR integration as "pre" and those encounters after the integration as "post". This allows us to quantify the changes associated with the integration. |
| Statistical methods | (*a*) Describe all statistical methods, including those used to control for confounding | A total of six fixed-effects and six random-effects feasible generalized least squares regressions were run with the source of specialist care referral as the grouping variable (i.e., the source of each primary care clinic). These grouping will ensure that any primary care clinic difference can be controlled. These regressions were performed with outcome variables 1 to 6 (see Section "Variables" above).<br><br>A separate fixed and a random-effects logistic regression were run with the binary outcome variable 7 (Section "Variable" above)<br><br>To control for any confound the estimations involved using medical specialty of the specialist clinic, the ICD-10 problem code, the procedure performed, the case's urgency as assessed by the primary care, the patient's gender, race, marital status, and visit date as control variables. |
| | (*b*) Describe any methods used to examine subgroups and interactions | The objective of this study is not to examine the differences across the six primary care clinics. The study control for the potential difference of these clinics but did not observe any significant differences across them for our various key outcome variables. |
| | (*c*) Explain how missing data were addressed | Missing data occurred for patient bookings where they did not come for the appointment (no-show) |
| | (*d*) If applicable, explain how loss to follow-up was addressed | Unfortunately, patients who do not show up for the appointment are omission biases that cannot be measured. |
| | (*e*) Describe any sensitivity analyses | For each outcome variable to be examined, two models (one fixed-effects and one |

| | | |
|---|---|---|
| | | random-effects) were run. This ensures that the results obtained are consistent across different estimation modes. |
| **Results** | | |
| Participants | (a) Report numbers of individuals at each stage of study—eg numbers potentially eligible, examined for eligibility, confirmed eligible, included in the study, completing follow-up, and analysed | 25404 referral encounters were extracted and 22309 have complete data where the patient completed the consultation session. |
| | (b) Give reasons for non-participation at each stage | Patient no-shows for specialist appointments could be a result of seeking alternative treatment avenues. |
| | (c) Consider use of a flow diagram | Data censoring only occurred at one stage, hence, we report these statistics as in line text instead. |
| Descriptive data | (a) Give characteristics of study participants (eg demographic, clinical, social) and information on exposures and potential confounders | Refer to Table 2a within the manuscript |
| | (b) Indicate number of participants with missing data for each variable of interest | A total of 22,309 encounters have complete data — 3,095 encounters do not have complete data as patients did not attend/ complete the specialist appointment that was referred to by the primary care health institution. |
| | (c) Summarise follow-up time (eg, average and total amount) | It is not applicable as data is retrospectively collected from the electronic medical records. |
| Outcome data | Report numbers of outcome events or summary measures over time | Refer to Table 2b within the manuscript |

| | | |
|---|---|---|
| Main results | (*a*) Give unadjusted estimates and, if applicable, confounder-adjusted estimates and their precision (eg, 95% confidence interval). Make clear which confounders were adjusted for and why they were included | Refer to Table 3a, Table 3b in the manuscript.<br>These estimates adjust for various confounds including:<br>Medical specialty of the specialist clinic, the ICD-10 problem code, the procedure performed, the case's urgency as assessed by the primary care, the patient's gender, race, marital status, and visit date as well as the source of the referral. |
| | (*b*) Report category boundaries when continuous variables were categorized | No continuous variables were categorized. |
| | (*c*) If relevant, consider translating estimates of relative risk into absolute risk for a meaningful time period | Not applicable. |
| Other analyses | Report other analyses done—eg analyses of subgroups and interactions, and sensitivity analyses | Not applicable. |
