# Supplementary material for: The Benefits of Integrating Electronic Medical Record Systems Between Primary and Specialist Care Institutions: Mixed Methods Cohort Study
Source: J Med Internet Res. 2025 Apr 22;27:e49363. doi: 10.2196/49363 (PMC12056414; doi:10.2196/49363)
Supplement: Multimedia Appendix 2 [file jmir_v27i1e49363_app2.pdf]

COREQ

Domain 1: Research team and reflexivity

1. One author/researcher and a research associate conducted the interviews.
2. The author/researcher has a PhD in Business/Information Systems and the research associate has a Master in linguistics.
3. The author/researcher is an associate professor and a faculty in a business school and the research associate was employed as part of the research team.
4. The author/researcher is male while the research associate is female.
5. The author/researcher is trained in qualitative research methods while the research associate is trained in interview method.
6. The author/researcher and the research associate had no prior relationship with the interviewees
7. The participants/interviewees had no prior knowledge of the project.
8. The author/researchers had no specific bias or assumptions of the research topic. The author/researcher was invited to study the project by the project sponsors but had not vested interest in the project.

Domain 2: Study design

9. Framework: The study followed basic tenets of a grounded theory approach and was guided partly by the research question – which is the impact of the new EMR system on the referral process.

Participant selection:

10. Sampling: Interviewees were selected based on purposive sampling. We selected interviewees based on their roles and organizational units – we selected those who worked in the specific specialist clinics (chosen for the high volume of referrals received from the primary care clinics), the call center, and in specific primary care clinics (chosen for sending high number of referrals to the specialist clinics). Within those clinics and call center, we selected those who were held clinical and non-clinical roles and were involved in the referral process (from writing the referral, to processing, scheduling, and receiving the referrals). We wanted to have a diverse set of interviewees involved in the referral process.
11. Method of approach: We approached the interviewees via email – the hospital and clinic managers had endorsed our email invitation prior to sending the invitation to the interviewees.
12. Sample size and non-participation: We had 30 interviewees in our study.
13. Non-participation: There were no dropouts.

Setting

14. Setting: We conducted our interviews at the interviewees' office and via online Zoom.
15. Presence of non-participants: There nobody else present except for the interviewee and the researchers.
16. Description of sample: The key characteristic of the sample was their role in the referral process as described in the sampling approach.

Data collection

17. Interview guide: We developed a basic interview guide with a set of standardized interview questions. However, we allowed the interviewees to guide the interview depending on their responses and we did not strictly follow the guide.
18. Repeat interviews: We did not conduct any repeat interviews.
19. Audio recording: We recorded (with consent) the interviews. We did not collect any video recordings.
20. Field notes: We made field notes during and after the interviews.
21. Duration: Most of our interviews were about 30 minutes to an hour in duration.
22. Data Saturation: We did not discuss data saturation. We recruited as many interviewees as possible from the sites.
23. Transcripts: We did not return our transcripts for comment or correction.

Domain 3: Analysis and findings

24. Number of data coders: There were two data coders.
25. Description of coding tree: The coding structure was provided in the manuscript. We coded the data by organizational unit (primary care clinic vs. specialist clinic).
26. Themes: For each unit, we derived emergent themes from the data and we compared that with the data derived from other dataset (survey). The themes were thus derived from a combination of emergent data themes with the survey themes.
27. Software: We used MaxQDA (version 2020) to manage our data and data analysis.
28. Participant checking: We presented our narrative findings to the key stakeholders from the specialist clinic and primary care clinic for validation.
29. Quotations: We did present interviewee quotations to illustrate the themes and findings. Each quotation was identified by the title of the interviewee.
30. Data and findings: There was consistency in our data and findings.
31. Clarity of major themes: The major themes ("patient diagnosis tracking", "patient coordination", and "patient care management") were presented in the findings.
32. Clarify of minor themes: There were no descriptions of diverse/minor cases or themes.
